# Screening of osteoarthritis diagnostic markers based on immune-related genes and immune infiltration

**DOI:** 10.1038/s41598-021-86319-7

**Published:** 2021-03-29

**Authors:** Wen-Hua Yuan, Qi-Qi Xie, Ke-Ping Wang, Wei Shen, Xiao-Fei Feng, Zheng Liu, Jin-Tao Shi, Xiao-Bo Zhang, Kai Zhang, Ya-Jun Deng, Hai-Yu Zhou

**Affiliations:** 1 Department of Orthopaedics, Xichang People’s Hospital, Xichang, 615000 Sichuan People’s Republic of China; 2 Department of Orthopaedics, Lanzhou University Second Hospital, Lanzhou, 730000 Gansu People’s Republic of China; 3 Lanzhou University, Lanzhou, 730000 Gansu People’s Republic of China; 4 Key Laboratory of Orthopaedics Disease of Gansu Province, Lanzhou, 730000 Gansu People’s Republic of China; 5 Department of Orthopaedics, Xigu District People’s Hospital of Lanzhou City, Lanzhou, 730000 Gansu People’s Republic of China; 6 Breast Disease Diagnosis and Treatment Center of Affiliated Hospital of Qinghai University & Affiliated Cancer Hospital of Qinghai University, Xining, 810000 Qinghai People’s Republic of China

**Keywords:** Computational biology and bioinformatics, Immunology, Biomarkers, Diseases

## Abstract

Osteoarthritis (OA) is a chronic degenerative disease of the bone and joints. Immune-related genes and immune cell infiltration are important in OA development. We analyzed immune-related genes and immune infiltrates to identify OA diagnostic markers. The datasets GSE51588, GSE55235, GSE55457, GSE82107, and GSE114007 were downloaded from the Gene Expression Omnibus database. First, R software was used to identify differentially expressed genes (DEGs) and differentially expressed immune-related genes (DEIRGs), and functional correlation analysis was conducted. Second, CIBERSORT was used to evaluate infiltration of immune cells in OA tissue. Finally, the least absolute shrinkage and selection operator logistic regression algorithm and support vector machine-recurrent feature elimination algorithm were used to screen and verify diagnostic markers of OA. A total of 711 DEGs and 270 DEIRGs were identified in this study. Functional enrichment analysis showed that the DEGs and DEIRGs are closely related to cellular calcium ion homeostasis, ion channel complexes, chemokine signaling pathways, and JAK-STAT signaling pathways. Differential analysis of immune cell infiltration showed that M1 macrophage infiltration was increased but that mast cell and neutrophil infiltration were decreased in OA samples. The machine learning algorithm cross-identified 15 biomarkers (BTC, PSMD8, TLR3, IL7, APOD, CIITA, IFIH1, CDC42, FGF9, TNFAIP3, CX3CR1, ERAP2, SEMA3D, MPO, and plasma cells). According to pass validation, all 15 biomarkers had high diagnostic efficacy (AUC > 0.7), and the diagnostic efficiency was higher when the 15 biomarkers were fitted into one variable (AUC = 0.758). We developed 15 biomarkers for OA diagnosis. The findings provide a new understanding of the molecular mechanism of OA from the perspective of immunology.

## Introduction

Osteoarthritis (OA) is a degenerative disease characterized by a reduction in articular cartilage tissue, thickening of the subchondral bone, and formation of osteophytes^1^. Some studies suggest that by detecting cartilage degradation in OA, biomarkers provide useful diagnostic information, reflecting disease-relevant biological activity and predicting the course of disease progression^2^. Therefore, exploring diagnostic biomarkers for OA may have important clinical applications.

In recent years, an increasing number of studies have demonstrated that immune cell infiltration plays an important role in OA development. For example, it has been shown that CD4+ T cells are significantly infiltrated in OA joints and that CD4+ T cells promote the polarization of activated Th1 cells and increase the secretion of immune regulatory cytokines. This local inflammation further aggravates the OA process^3^. Another study showed that OA joints have obvious immune cell infiltration, including CD14+ macrophages, CD4+ T cells, CD8+ T cells and CD16+ CD56+ natural killer cells^4^. Therefore, from the perspective of the immune system, evaluating the infiltration of immune cells and determining differences in the composition of infiltrating immune cells will help in the development of new diagnostic biomarkers and immunotherapeutic targets.

CIBERSORT is a method to describe the composition of immune cells in complex tissues based on their gene expression profiles^5^. To date, few studies have used CIBERSPORT to analyze immune cell infiltration in OA. In this study, we analyzed differentially expressed immune-related genes and immune cell infiltration using microarray data from patients with OA and normal control subjects. Machine learning algorithms were applied to further identify diagnostic biomarkers of OA.

## Methods

### Data download

Gene Expression Omnibus (GEO, https://www.ncbi.nlm.nih.gov/geo/)^6^ is an international public repository for the storage and free distribution of microarrays, second-generation sequencing, and other forms of high-throughput functional genomic data sets. We used the R language GEOquery package^7^ to download sample-derived reliable OA expression profiling datasets from the GEO datasets GSE51588^8^, GSE55235^9^, GSE55457^9^, GSE82107^10^, and GSE114007^11^. Expression data types from *Homo sapiens* were subjected to expression profiling by array. The GSE51588 dataset is based on the GPL13497 platform Agilent–026652 human whole-genome microarray (Agilent Technologies, Santa Clara, CA, USA) and includes 40 cases of OA subchondral bone tissue and 10 normal subchondral bone tissue samples. The GSE55235 and GSE55457 datasets are based on the GPL96 platform [HG-U133A] Affymetrix Human Genome U133A Array (Santa Clara, CA, USA). GSE55235 includes 10 OA synovial tissue samples, 10 rheumatoid arthritis (RA) tissue samples, and 10 normal human synovial tissue samples; we analyzed 10 OA and 10 normal synovial tissue samples from this dataset. GSE55457 includes 10 OA synovial tissue samples, 13 RA tissue samples, and 10 normal human synovial tissue samples; we analyzed 10 OA and 10 normal synovial tissue samples from this dataset. GSE82107 is based on the GPL570 platform [HG-U133_Plus_2] Affymetrix Human Genome U133 Plus 2.0 array and includes 10 OA synovial tissue samples and 7 normal human synovial tissue samples, all of which were analyzed in this study. GSE114007 is based on the GPL18573 platform Illumina NextSeq 500 and includes 20 OA cartilage tissue samples and 18 normal human cartilage tissue samples, all of which were examined in this study.

### Data processing and screening of differentially expressed genes (DEGs)

We used the affy package^12^ with R language (version 3.6.1, http://r-project.org/) to process the raw data of the GSE51588, GSE55235, GSE55457, GSE82107, and GSE114007 datasets, which were subjected to background correction and data normalization using the RMA algorithm. The gene annotation file corresponding to the Bioconductor (http://www.bioconductor.org/)^13^ platform was used to annotate the probe matrix. The expression matrices of the GSE51588, GSE55235, GSE55457, and GSE114007 datasets were merged, and interbatch differences were removed using the sva package^14^. The effect of removing interbatch differences was visualized using a quantile–quantile plot (Q-Q plot), and the effect of intersample correction was displayed using a two-dimensional PCA cluster plot. The R language limma package^15^ was employed to perform differential expression analysis and output DEGs. DEGs satisfied an adjusted P value < 0.05 and |log2-fold-change|> 1. To visually display the DEGs, volcano plots were drawn using the ggplot2 package^16^.

### GO analysis, KEGG pathway enrichment analysis, and PPI network analysis of DEGs

GO covers three aspects of biology: BP, CC, and MF^17^. KEGG is a database for understanding the high-level functions of biological systems at the molecular level^18^. To further analyze the functions of the DEGs, they were analyzed by GO and KEGG pathway enrichment analyses using the R language Clusterprofile package^19^, and P < 0.05 was considered to indicate a statistically significant difference. We built a PPI network using the STRING database (version 11.0, http://www.string-db.org/)^20^. Cytoscape software (version 3.7.1)^21^ was used to visualize the PPI network, and the cytoHubba plug-in^22^ was employed to select the top 10 DEGs in the maximum correlation criterion (MCC) as hub genes.

### Extraction of immune-related genes, screening, and functional analysis of differentially expressed immune-related genes (DEIRGs)

Immunology Database and Analysis Portal (ImmPort)^23^ is a comprehensive database that collates immune-related genes directly involved in immune-related processes from research papers, books, electronic resources, etc., aiming to provide analytical tools for studies of basic and clinical immunology. We downloaded and collated immune-related genes from this database, extracted the expression matrix of immune-related genes using R language, and performed differential expression analysis with the limma package to identify DEIRGs. The DEIRGs satisfied an adjusted P value < 0.05 and |log2-fold-change| >  1. We then used the ggplot2 package to draw a volcano plot of the DEIRGs. After GO and KEGG pathway enrichment analyses of the DEIRGs using the R software clusterProfile package, statistical significance was determined based on P < 0.05. We constructed a PPI network related to DEIRGs using the STRING database, and Cytoscape software was used to visualize the network. The cytoHubba plugin was used to select the top 10 DEIRGs from the MCC as hub immune-related genes. To explore the similarity between hub immune-related genes, we applied the GOSemSim package^24^ to score the geometric mean of the semantic similarity of 10 hub immune-related genes in BP, CC, and MF categories to detect similarities in protein functions.

### Evaluation, analysis, and visualization of immune cell infiltration

CIBERSORT (https://cibersort.stanford.edu/) is a tool for deconvolution of the expression matrix of immune cell subtypes based on the principle of linear support vector regression^5^. We uploaded the previously obtained gene expression matrix data to CIBERSORT and filtered samples showing P < 0.05. The R language corrplot package^25^ was used to plot a correlation heat map to visualize the correlation of 22 types of immune cell infiltrates. The igraph package (https://github.com/igraph/rigraph) was used to draw a correlation network diagram of immune cell infiltration to visualize the interactions of the 22 types of infiltrating immune cells, with P < 0.05 and |correlation coefficient| >  0.15 as the interaction standards. We utilized the ggplot2 package to draw a violin plot to visualize differences in infiltration of the 22 types of immune cells.

### Screening and validation of diagnostic markers

SVM-RFE is a machine learning method based on support vector machines used to find the best variables by deleting feature vectors produced by SVM^26^. LASSO logistic regression is a machine learning method for determining variables by finding the λ with the smallest classification error^27^. The two algorithms are mainly used to screen feature variables and construct the best classification model. We extracted DEIRG expression matrices, merged them with 22 types of immune cell matrices, and then simultaneously screened biomarkers for OA using the above two algorithms, after which we validated the resulting biomarkers for diagnostic efficacy based on the GSE82107 dataset.

## Results

### Data preprocessing, data filtering, and DEG identification

**Figure 1:**
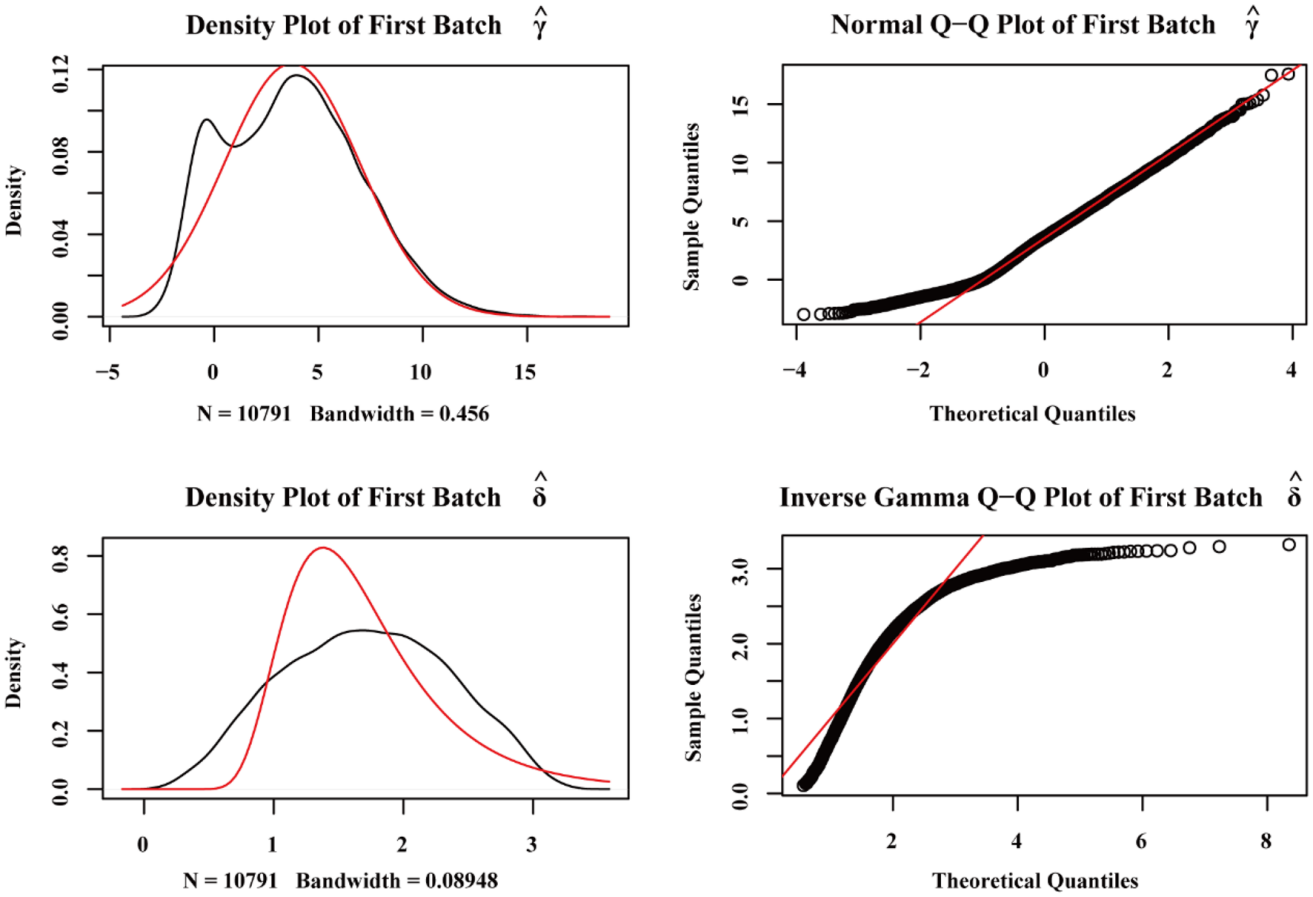
Q-Q chart of data set eliminating inter-batch differences.

**Figure 2:**
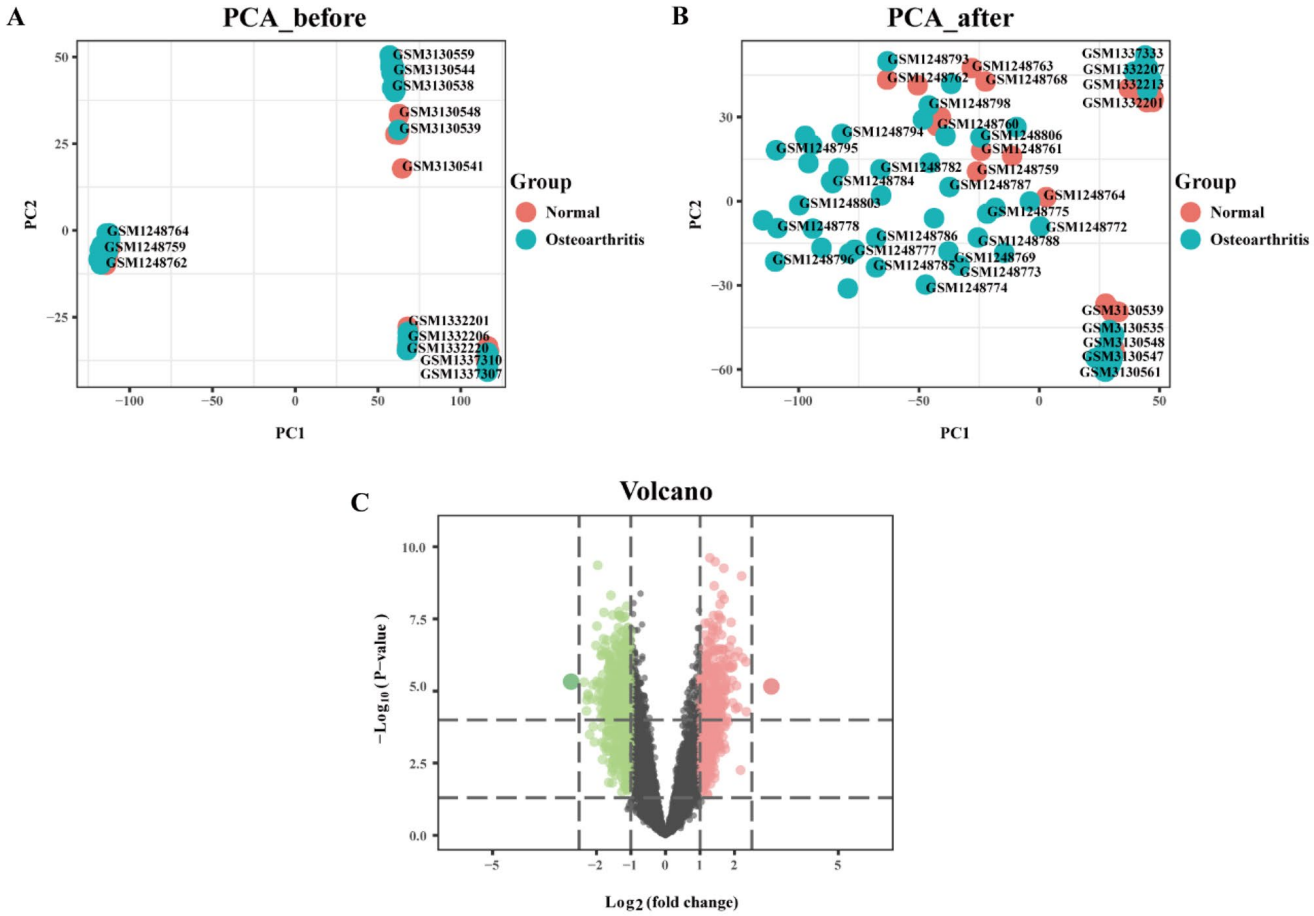
Two-dimensional PCA cluster plot before and after sample correction and volcano plots of DEGs. (**A**,**B**) Figure shows two-dimensional PCA cluster plots before and after correcting for inter-batch differences removed for GSE51588, GSE55235, GSE55457, and GSE114007, respectively. Blue represents the osteoarthritis group and red represents the normal control group. (**C**) DEG volcano plot; red represents up-regulated differentially expressed genes and green represents down-regulated differentially expressed genes.

We first merged the gene expression matrix data for the GSE51588, GSE55235, GSE55457, and GSE114007 datasets, removed differences between batches, and constructed a Q-Q plot (Fig. 1). The results showed that interbatch differences between samples had been removed. The merged matrix was standardized and processed. Before and after standardization, a two-dimensional principal component analysis (PCA) clustering diagram was generated (Fig. 2A,B). The results showed that the samples in the OA and normal control groups were clearly clustered after standardization, indicating that the sample sources were reliable. After data preprocessing, we extracted 711 DEGs from the expression matrix using R language, and the results are shown in a volcano plot (Fig. 2C).

### GO analysis, KEGG enrichment analysis, and PPI network analysis of DEGs

**Figure 3:**
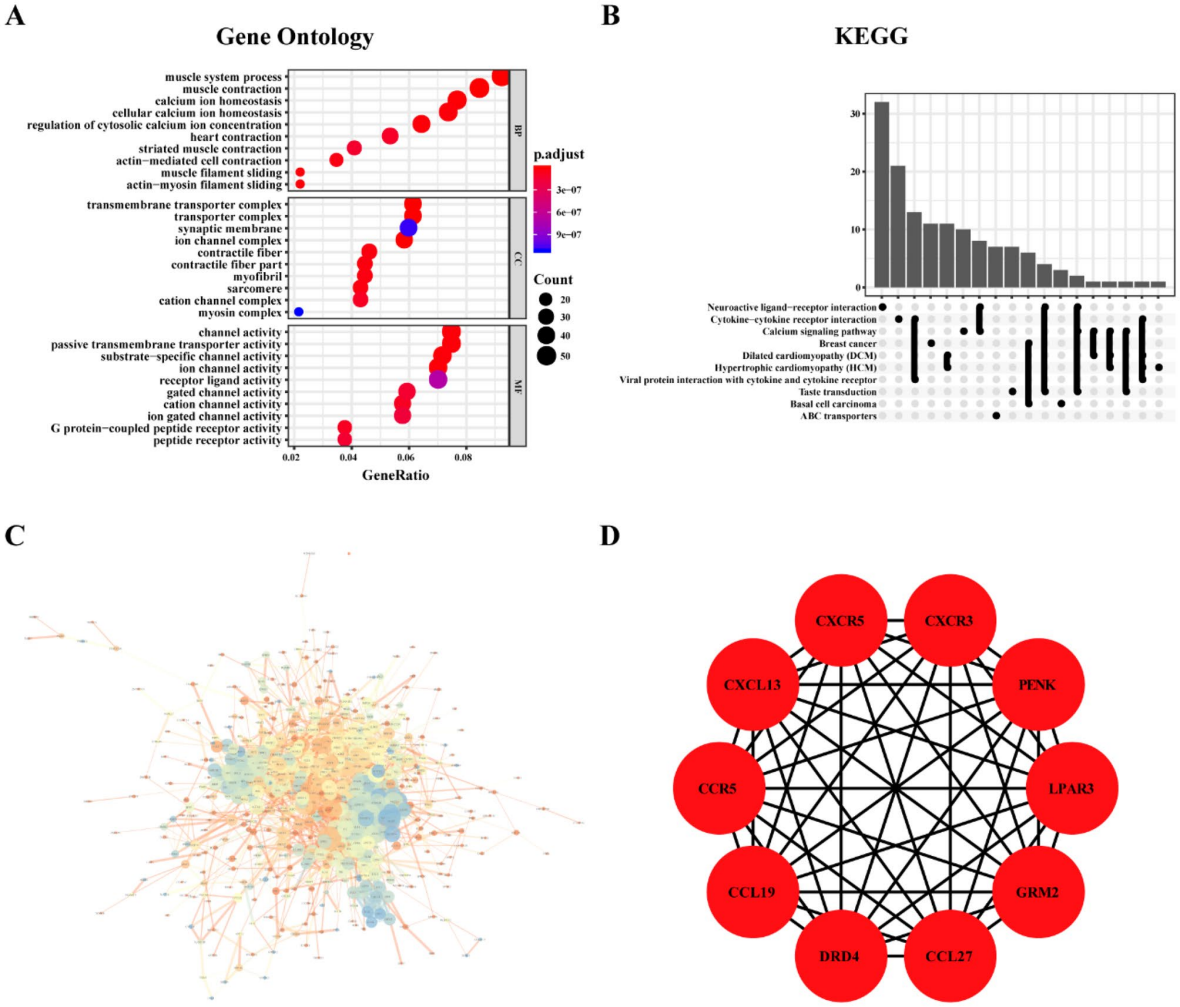
GO, KEGG, and PPI network analyses of DEGs. (**A**) GO biological function enrichment analysis. (**B**) KEGG pathway enrichment analysis. (**C**) PPI network analysis graph. The node size indicates the clustering coefficient; a larger node indicated a larger clustering coefficient, with a greater proportion of genes in the network. The node color indicates the degree; a higher degree indicated greater connectedness of the node. Blue represents a larger degree, yellow indicated a medium degree, and orange indicates a minimum degree. The thickness of the line represents the overall score. A higher score resulted in a thicker line, indicating a strong interaction between the two proteins. (**D**) Schematic representation of hub genes.

The Gene Ontology (GO) analysis results showed that for biological process (BP), the DEGs were significantly enriched in the muscle system process, calcium ion homeostasis, and cellular calcium ion homeostasis. The cellular component (CC) category mainly showed enrichment in the synaptic membrane, ion channel complex, and contractile fiber and the molecular function (MF) category mainly in channel activity and ion channel activity (Fig. 3A). The results of Kyoto Encyclopedia of Genes and Genomes (KEGG) pathway enrichment analysis revealed enrichment mainly in neuroactive ligand-receptor interactions, cytokine-cytokine receptor interactions, and calcium signaling pathways (Fig. 3B). The protein–protein interaction (PPI) network constructed by STRING is depicted in Fig. 3C, and the 10 hub genes selected using the cytoHubba plugin were *CXCR5*, *CXCL13*, *CCL19*, *LPAR3*, *CXCR3*, *CCR5*, *CCL27*, *GRM2*, *DRD4*, and *PENK* (Fig. 3D).

### Identification and functional similarity analysis of DEIRGs

**Figure 4:**
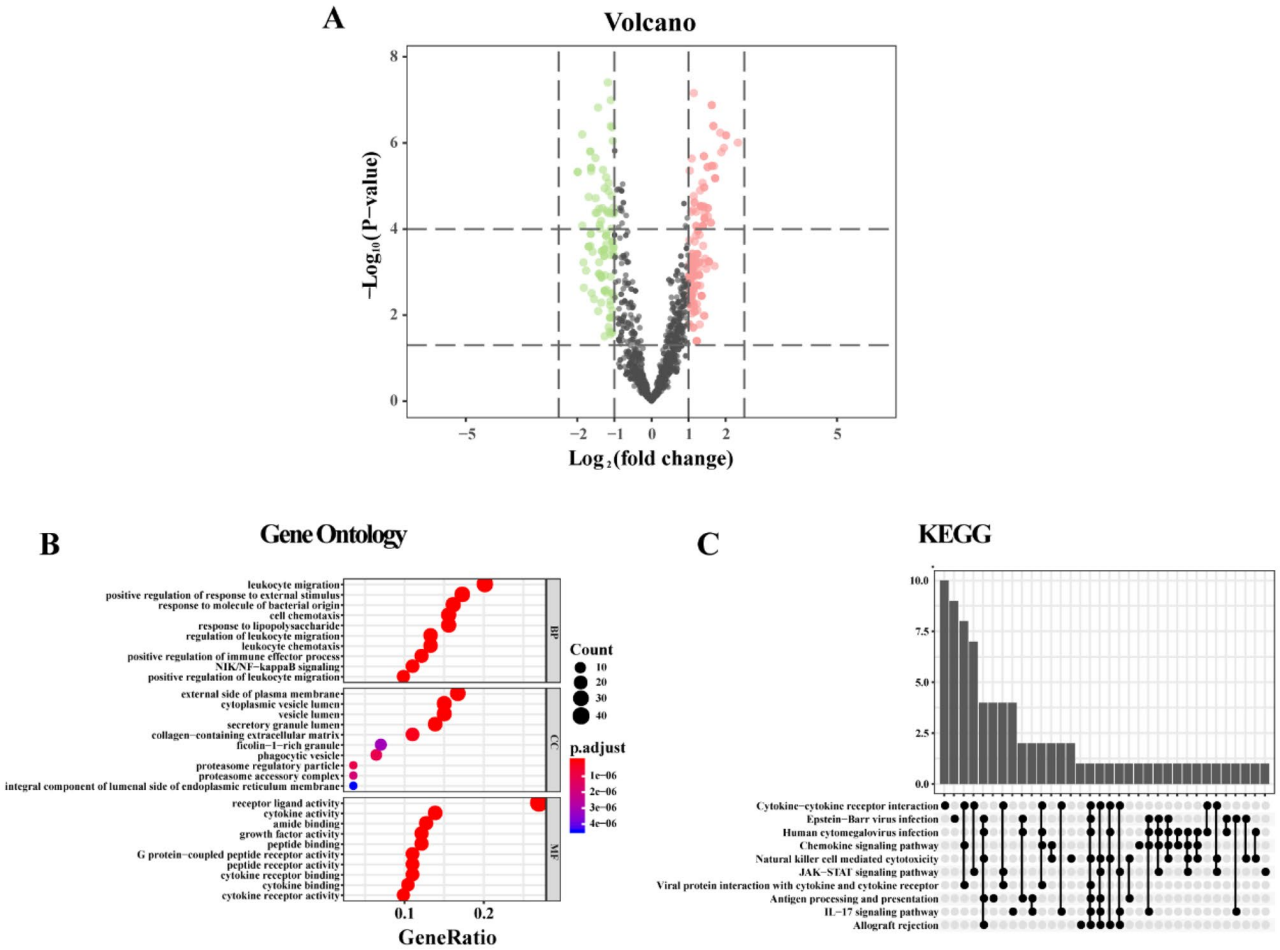
Volcano plot of DEIRGs and GO and KEGG enrichment analysis. (**A**) Volcanic map of DEIRGs; red represents up-regulation of these genes, whereas green represents down-regulation of these genes; (**B**) GO biological function enrichment analysis; C. KEGG pathway enrichment analysis.

**Figure 5:**
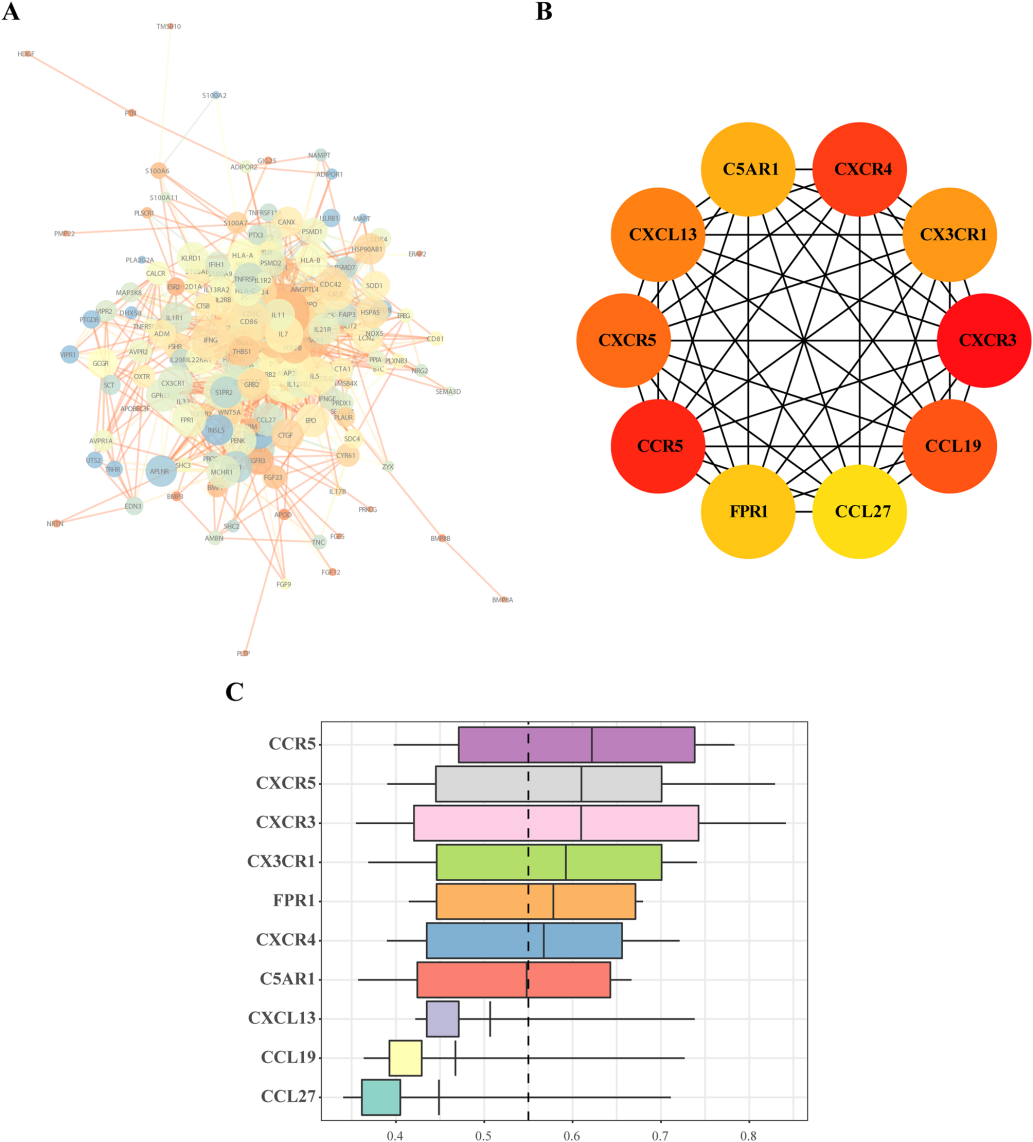
PPI network analysis and functional similarity analysis of DEIRGs. (**A**) PPI network analysis graph, the node size indicates the clustering coefficient; a larger node indicates a larger the clustering coefficient, and thus a greater proportion of genes in the network. The node color indicates the degree; a higher degree indicates greater connectedness of the node. Blue represents a higher degree, yellow represents a medium degree, and orange indicates a minimum degree. The line thickness represents the overall score. A higher score results in a thicker line, indicating that the interaction between the two proteins is stronger. (**B**) Schematic representation of hub immune-related genes. (**C**) Hub immune-related gene similarity analysis plot, with the abscissa as the similarity score.

After extracting the immune-related gene expression matrix, we identified 270 DEIRGs using R language, and the results are illustrated in a volcano plot (Fig. 4A). According to GO analysis, the DEIRGs were significantly enriched in BP categories leukocyte migration, positive regulation of response to external stimulus, response to molecule of bacterial origin, and cell chemotaxis. CC categories mainly showed enrichment of the external side of the plasma membrane, cytoplasmic vesicle lumen, and vesicle lumen; MF categories mainly included receptor ligand activity and cytokine activity (Fig. 4B). KEGG pathway enrichment analysis showed enrichment mainly in cytokine-cytokine receptor interactions, chemokine signaling pathways, natural killer cell-mediated cytotoxicity, and JAK-STAT signaling pathways (Fig. 4C). The PPI network of DEIRGs constructed by STRING is shown in Fig. 5A. The 10 hub immune-related genes selected using the cytoHubba plugin were *CXCR3*, *CCR5*, *CXCR4*, *CCL19*, *CXCR5*, *CXCL13*, *CX3CR1*, *C5AR1*, *FPR1*, and *CCL27* (Fig. 5B). Functional similarity analysis results revealed high similarity scores for *CCR5*, *CXCR5*, *CXCR3*, *CX3CR1*, *FPR1*, and *CXCR4* (Fig. 5C).

### Immune cell infiltration analysis results

**Figure 6:**
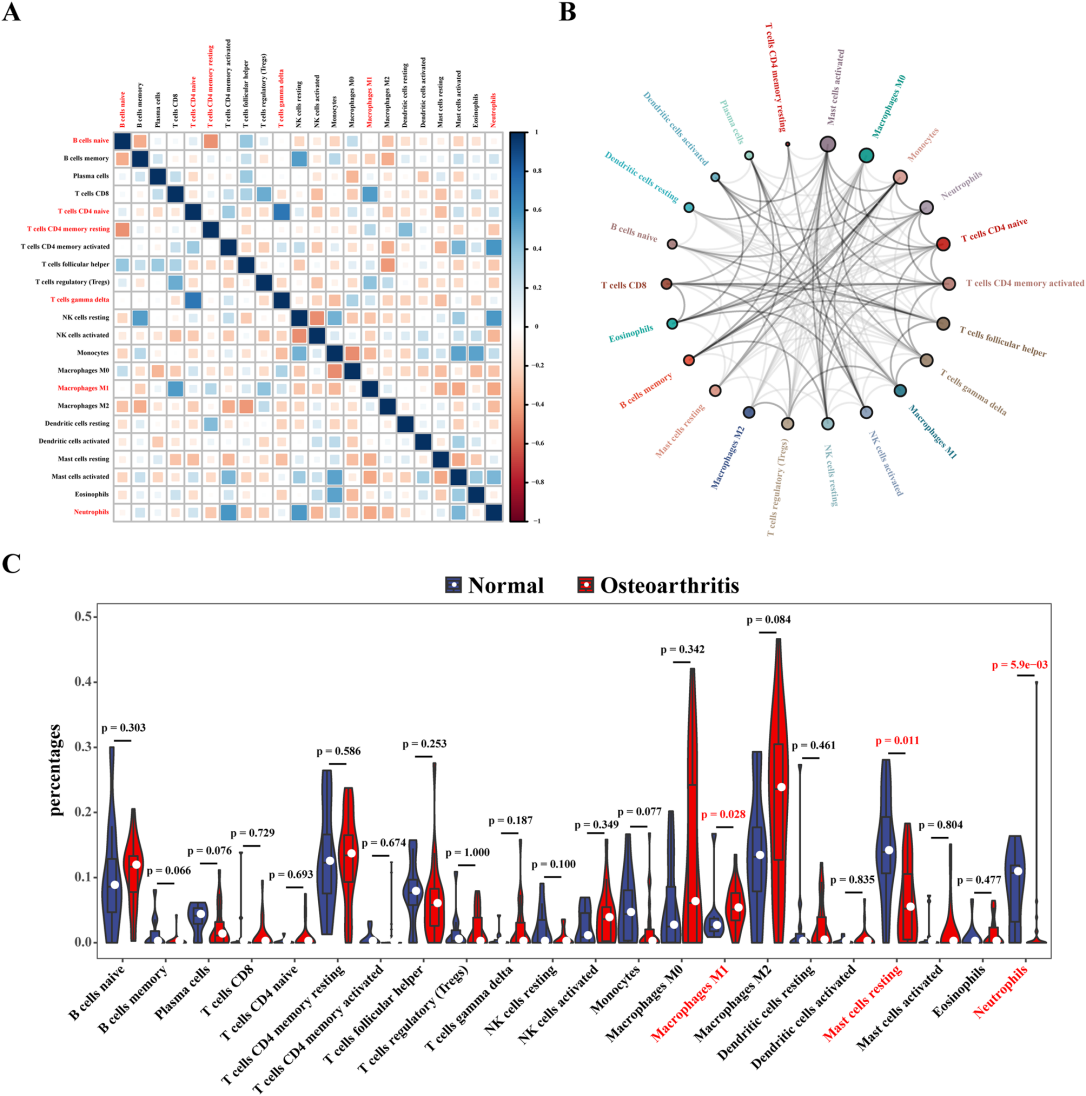
Correlation plots of immune cell infiltration analysis. (**A**) Correlation heat map of 22 immune cells. Blue represents positive correlation, red represents negative correlation, the darker the color, the stronger the correlation. (**B**) Network diagram of interactions of 22 types of immune cells. The size of the circle represents the interaction strength between immune cells infiltrating cells. (**C**) Violin plot of the proportion of infiltration by 22 types of immune cells in normal control samples versus in osteoarthritis samples. Red markers represent differences in infiltration between the two groups of samples.

The correlation heat map showed that (Fig. 6A) neutrophils correlated positively with CD4 activated memory T cells (r = 0.59, *p* < 0.05) and resting natural killer (NK) cells (r = 0.57, *p* < 0.05), gamma delta T cells correlated positively with CD4 naïve T cells (r = 0.73, *p* < 0.05), M0 macrophages correlated negatively with monocytes (r = − 0.46, *p* < 0.05), and naïve B cells correlated negatively with CD4 memory resting T cells (r = − 0.45, *p* < 0.05). The immune cell interaction network illustrated that activated M0 mast cells and monocytes have a strong relationship with other immune cells but that T memory resting CD4 cells and plasma cells have a weak relationship with other immune cells (Fig. 6B). As shown in Fig. 6C, the degree of M1 macrophage infiltration was higher than in normal samples (*p* < 0.05), though the degree of resting mast cell and neutrophil infiltration was lower in OA samples (*p* < 0.05).

### Screening and validation of OA biomarkers

**Figure 7:**
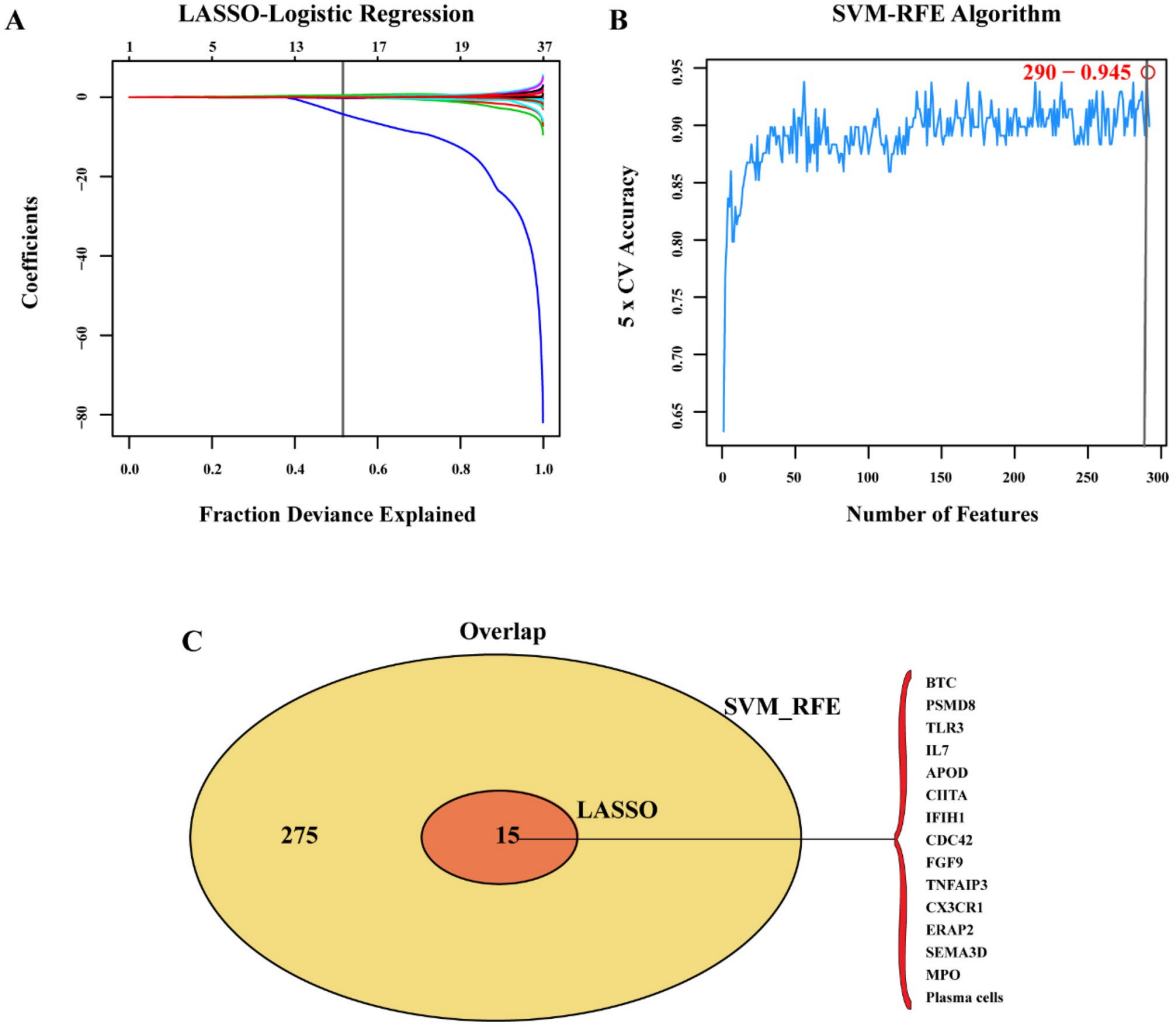
Biomarker selection was performed using two algorithms. (**A**) LASSO logistic regression algorithm to screen for biomarkers; (**B**) SVM-RFE algorithm for screening biomarkers; (**C**) Venn diagram showing the intersection of biomarkers obtained by the two algorithms.

**Figure 8:**
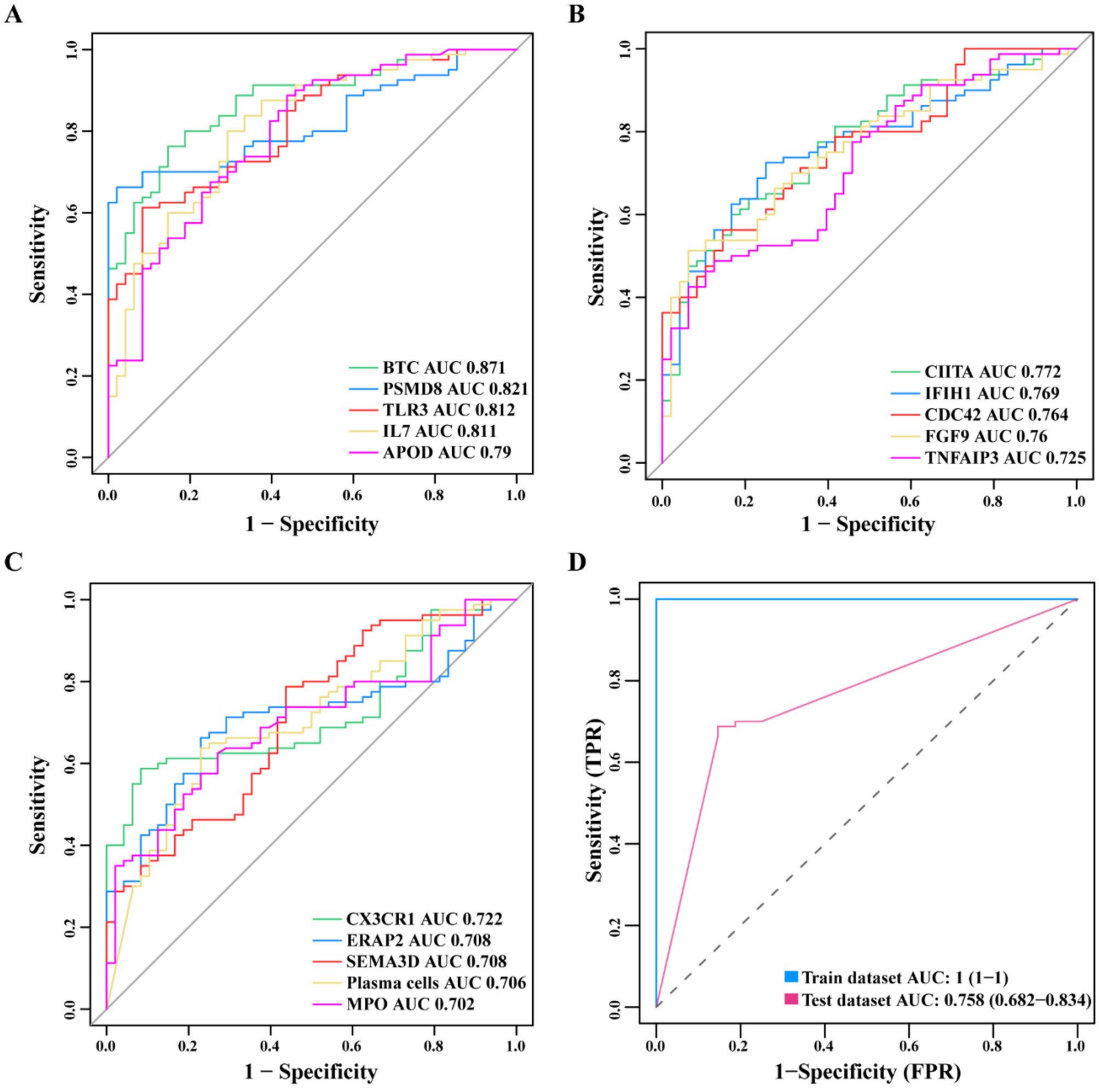
Verification of biomarkers. (**A**–**C**) Validation of the diagnostic efficacy of 15 biomarkers in the validation set; (**D**) validation of diagnostic efficacy after fitting 15 biomarkers into one variable.

To screen for biomarkers of OA, we combined DEIRGs with the matrices of 22 types of immune cells. First, 15 biomarkers were identified using the least absolute shrinkage and selection operator (LASSO) logistic regression algorithm (Fig. 7A). Next, we used the support vector machine-recurrent feature elimination (SVM-RFE) algorithm to screen 290 biomarkers (Fig. 7B). Taking the intersecting results from the two approaches, the results (Fig. 7C) showed *BTC*, *PSMD8*, *TLR3*, *IL7*, *APOD*, *CIITA*, *IFIH1*, *CDC42*, *FGF9*, *TNFAIP3*, *CX3CR1*, *ERAP2*, *SEMA3D*, *MPO*, and plasma cells to be potential biomarkers. To verify the diagnostic efficacy of biomarkers, we verified the above 15 biomarkers using the GSE82107 dataset as the validation set. Figures 8A–C show that all 15 biomarkers had high diagnostic efficacy (AUC > 0.7). When 15 biomarkers were fitted into one variable, the diagnostic efficiency was 1 in the training set and was higher in the validation set (AUC = 0.758). The specific results are presented in Fig. 8D.

## Discussion

OA is a chronic joint disease characterized by progressive degeneration of articular cartilage, and the pathogenesis of OA is not completely understood^28^. Research shows that immune cell infiltration plays an important role in the development of OA^3,4^. In addition, biomarkers reflect changes at the molecular level and can accurately monitor pathological changes in articular cartilage and provide important information for the diagnosis of OA^2,29^. Therefore, analyzing the pattern of OA immune cell infiltration and finding specific diagnostic markers have profound significance for OA patients. With the rapid development of science and technology, bioinformatics provides a powerful strategy for the screening of molecular markers, and the CIBERSPORT tool also facilitates analysis of immune cell infiltration patterns of diseases. In this study, we employed CIBERSORT to remine expression spectrum data of OA in the GEO database, analyze the pattern of OA immune cell infiltration, and simultaneously integrate and analyze immune-related genes, and we used a machine learning algorithm to screen for diagnostic biomarkers of OA.

A total of 711 DEGs were identified. GO analysis results showed that the DEGs are closely related to cellular calcium ion homeostasis, ion channel complexes, channel activity and ion channel activity. Boileau et al. reported that by inhibiting the Erk1/2 signaling pathway, which inhibits the induction of major catabolic factors during OA cartilage degradation, the calcium signaling pathway is involved in the development of OA^30^.

We also constructed a PPI network of the DEGs and identified 10 hub genes. One study found that CXCL13 was highly expressed in OA samples and that CXCL13 may directly modulate cellular proliferation and collagen type I in OA patients, contributing to the remodeling process that occurs in the evolution of the disease^31^. In addition, Haringman et al. used immunohistochemistry to detect synovial tissue of OA patients and found CCR5 to be highly expressed^32^. Another study reported that CXCR3 is a key link for neutrophils and NK cells to promote the progression of OA^33^. In conclusion, previous studies support our results, suggesting that CXCL13, CCR5 and CXCR3 play an important role in OA. Nevertheless, the roles of CCL19, LPAR3, CCL27, GRM2, DRD4 and PENK in OA remain unclear and deserve further study.

We then identified 270 DEIRGs. GO and KEGG analysis results for DEIRGs were similar to those for DEGs. Previous studies have shown that leukocyte migration is closely related to OA development, which is consistent with our results^33,34^. However, few studies of OA have evaluated cell-mediated cytotoxicity, which may play an important role in the occurrence and development of OA, and its mechanism should be further explored. In this study, functional enrichment analysis revealed significant enrichment of the JAK-STAT signaling pathway. In recent years, therapies targeting the immune checkpoint molecules programmed death ligand 1 (PD-L1) and indoleamine 2,3-dioxygenase 1 (IDO1) have been explored for various tumors^35^. A previous study showed that PD-L1 and IDO1 mRNA expression correlate positively with JAK2 and STAT1 mRNA expression. The results suggest the feasibility of combined inhibition of PD-L1 or IDO1 with JAK-STAT pathway inhibition to treat soft tissue leiomyosarcoma^36^. According to the above study, the JAK-STAT signaling pathway is closely related to the immune checkpoints PD-L1 and IDO1. Our analysis results show that the JAK-STAT signaling pathway may play an important role in the occurrence and progression of OA. Therefore, the development of new immunomodulatory agents for PD-L1 and IDO1 may contribute to the treatment of OA and help OA patients benefit from immunotherapy.

PPI analysis and functional similarity results revealed some genes with prominent roles in the network (*CX3CL1, CX3CR1, FPR1, CXCR4*). Hou et al. reported that *CX3CL1* activates *c-Raf, MEK, ERK*, and *NF-κB* at the *MMP-3* promoter through CX3CR1, leading to cartilage destruction during OA^37^. Nonetheless, the mechanisms of *FPR1* and *CXCR4* in OA remain unclear and require further investigation.

Our differential analysis of immune cell infiltration showed that M1 macrophage infiltration was increased and that mast cell and neutrophil infiltration were decreased in OA samples. Using enzymatic digestion, Bridges et al. isolated mast cells from the synovium of 48 patients with RA and 42 patients with OA, and the results suggested that activation of mast cells in the synovium of patients with clinically active OA, indicating their important role in OA progression and treatment^38^. One study found relatively high infiltration of mast cells into OA synovial tissue, as related to structural damage in patients with OA, suggesting that mast cells play an important role in OA^39^. Hua et al. reported that glucosamine may inhibit the function of neutrophils and thus may exhibit anti-inflammatory effects in arthritis^40^, and M1 macrophages in the synovium exacerbate experimental OA^41^. Previous studies support our results, suggesting that these immune cells play an important role in the progression of OA.

Furthermore, our results reveal the details of 22 types of immune cell infiltration in OA. Gamma delta T cells were closely related to naïve CD4 T cells, and neutrophils were closely related to activated memory CD4 T cells and resting NK cells. Additionally, activated mast cells and M0 macrophages most strongly interacted with other cells, whereas plasma cells showed the weakest interaction with other cells. The specific mechanism of action between these immune cells is still unclear, and further experimental exploration is still needed.

Finally, based on the matrix data of DEIRGs combined with 22 types of immune cells, 15 biomarkers (BTC, PSMD8, TLR3, IL7, APOD, CIITA, IFIH1, CDC42, FGF9, TNFAIP3, CX3CR1, ERAP2, SEMA3D, MPO, and plasma cells) were screened by cross-validation using LASSO logistic regression and the SVM-RFE algorithms. Steinbeck et al. identified and analyzed *MPO* in the synovial fluid of 4 cases of control (acute injury), 11 cases of early OA, and 18 cases of late OA^42^. The results showed that *MPO* can be used as a biomarker for early diagnosis of OA. In addition, Punzi et al. found that *MPO* may serve as a biomarker to facilitate the diagnosis of erosive hand OA^43^. These studies support the results of our analysis. However, no studies have reported the use of the other 14 biomarkers for the diagnosis of OA, which needs further research and verification. We also fitted 15 biomarkers into one variable for verification, which revealed high diagnostic efficacy in the training set (AUC = 1) and validation set (AUC = 0.758). Our results may contribute to the development of new tools and criteria for diagnosing and monitoring OA.

There were several limitations to this study. First, CIBERSORT is based on the principle of linear support vector regression and uses gene expression data in reverse to deduce the result of immune cell infiltration. Indeed, it is not based on experimental data, and further verification of immune cell infiltration by a large number of experiments is needed. Second, our data showed some heterogeneity. Although we performed quality control, homogenization, and standardization of the original data and also removed interbatch differences, a larger sample size and higher quality data set are needed to verify our results. Third, we performed mining and analysis of previously published data; although some previous studies showed similar results, the related molecules and their mechanisms at the molecular, cell, and tissue levels require validation.

In conclusion, we found that BTC, PSMD8, TLR3, IL7, APOD, CIITA, IFIH1, CDC42, FGF9, TNFAIP3, CX3CR1, EARP2, SEMA3D, MPO, and plasma cells can be used as diagnostic markers for OA. In addition, mast cells, neutrophils and M1 macrophages may play a key role in the occurrence and progression of OA. Further investigation of these immune cells may identify targets of immunotherapy for OA and help OA patients benefit from immunomodulatory therapy.

## References

[1] Varela-Eirin M (2018). Cartilage regeneration and ageing: Targeting cellular plasticity in osteoarthritis. Ageing Res. Rev..

[2] Mobasheri A (2012). Osteoarthritis year 2012 in review: Biomarkers. Osteoarthr. Cartil..

[3] Rosshirt N (2019). A predominant Th1 polarization is present in synovial fluid of end-stage osteoarthritic knee joints: Analysis of peripheral blood, synovial fluid and synovial membrane. Clin. Exp. Immunol..

[4] Moradi B (2015). Unicompartmental and bicompartmental knee osteoarthritis show different patterns of mononuclear cell infiltration and cytokine release in the affected joints. Clin. Exp. Immunol..

[5] Newman AM (2015). Robust enumeration of cell subsets from tissue expression profiles. Nat. Methods..

[6] Barrett T (2013). NCBI GEO: Archive for functional genomics data sets–update. Nucleic Acids Res..

[7] Davis S, Meltzer PS (2007). GEOquery: A bridge between the Gene Expression Omnibus (GEO) and BioConductor. Bioinformatics.

[8] Chou C-H (2013). Genome-wide expression profiles of subchondral bone in osteoarthritis. Arthritis Res. Ther..

[9] Woetzel D (2014). Identification of rheumatoid arthritis and osteoarthritis patients by transcriptome-based rule set generation. Arthritis Res. Ther..

[10] Broeren MGA (2016). Functional tissue analysis reveals successful cryopreservation of human osteoarthritic synovium. PLoS ONE.

[11] Fisch KM (2018). Identification of transcription factors responsible for dysregulated networks in human osteoarthritis cartilage by global gene expression analysis. Osteoarthr. Cartil..

[12] Gautier L, Cope L, Bolstad BM, Irizarry RA (2004). affy–analysis of Affymetrix GeneChip data at the probe level. Bioinformatics.

[13] Gentleman RC (2004). Bioconductor: Open software development for computational biology and bioinformatics. Genome Biol..

[14] Parker HS (2014). Preserving biological heterogeneity with a permuted surrogate variable analysis for genomics batch correction. Bioinformatics.

[15] Ritchie ME (2015). limma powers differential expression analyses for RNA-sequencing and microarray studies. Nucleic Acids Res..

[16] Ginestet C (2011). ggplot2: Elegant graphics for data analysis. J. R. Stat. Soc. A. Stat..

[17] Ashburner M (2000). Gene ontology: Tool for the unification of biology. The Gene Ontology Consortium. Nat. Genet..

[18] Kanehisa M, Goto S (2000). KEGG: Kyoto encyclopedia of genes and genomes. Nucleic Acids Res..

[19] Yu G, Wang L-G, Han Y, He Q-Y (2012). ClusterProfiler: An R package for comparing biological themes among gene clusters. OMICS..

[20] Szklarczyk D (2019). STRING v11: Protein-protein association networks with increased coverage, supporting functional discovery in genome-wide experimental datasets. Nucleic Acids Res..

[21] Shannon P (2003). Cytoscape: A software environment for integrated models of biomolecular interaction networks. Genome Res..

[22] Chin C-H (2014). CytoHubba: Identifying hub objects and sub-networks from complex interactome. BMC Syst. Biol..

[23] Bhattacharya S (2018). ImmPort, toward repurposing of open access immunological assay data for translational and clinical research. Sci. Data..

[24] Yu G (2010). GOSemSim: An R package for measuring semantic similarity among GO terms and gene products. Bioinformatics.

[25] Friendly M (2002). Corrgrams: Exploratory displays for correlation matrices. Am. Stat..

[26] Suykens JAK, Vandewalle J (1999). Least squares support vector machine classifiers. Neural Process. Lett..

[27] Tibshirani R (1996). Regression shrinkage and selection via the lasso. J. R. Stat. Soc. Ser. B Stat. Methodol..

[28] Väänänen T (2014). YKL-40 as a novel factor associated with inflammation and catabolic mechanisms in osteoarthritic joints. Mediators. Inflamm..

[29] Zanetti M, Bruder E, Romero J, Hodler J (2000). Bone marrow edema pattern in osteoarthritic knees: Correlation between MR imaging and histologic findings. Radiology.

[30] Boileau C (2006). PD-0200347, an alpha2delta ligand of the voltage gated calcium channel, inhibits in vivo activation of the Erk1/2 pathway in osteoarthritic chondrocytes: A PKCalpha dependent effect. Ann. Rheum. Dis..

[31] Lisignoli G (2006). CXCL12 (SDF-1) and CXCL13 (BCA-1) chemokines significantly induce proliferation and collagen type I expression in osteoblasts from osteoarthritis patients. J. Cell. Physiol..

[32] Haringman JJ, Smeets TJM, Reinders-Blankert P, Tak PP (2006). Chemokine and chemokine receptor expression in paired peripheral blood mononuclear cells and synovial tissue of patients with rheumatoid arthritis, osteoarthritis, and reactive arthritis. Ann. Rheum. Dis..

[33] Scanzello CR (2017). Chemokines and inflammation in osteoarthritis: Insights from patients and animal models. J. Orthop. Res..

[34] Wojdasiewicz P (2014). The chemokine CX3CL1 (fractalkine) and its receptor CX3CR1: Occurrence and potential role in osteoarthritis. Arch. Immunol. Ther. Exp.

[35] Ott PA, Hodi FS, Kaufman HL, Wigginton JM, Wolchok JD (2017). Combination immunotherapy: A road map. J. Immunother. Cancer..

[36] Iwasaki T (2020). Association of PD-L1 and IDO1 expression with JAK-STAT pathway activation in soft-tissue leiomyosarcoma. J. Cancer Res. Clin. Oncol..

[37] Hou S-M, Hou C-H, Liu J-F (2017). CX3CL1 promotes MMP-3 production via the CX3CR1, c-Raf, MEK, ERK, and NF-κB signaling pathway in osteoarthritis synovial fibroblasts. Arthritis Res. Ther..

[38] Bridges AJ (1991). Human synovial mast cell involvement in rheumatoid arthritis and osteoarthritis. Relationship to disease type, clinical activity, and antirheumatic therapy. Arthritis. Rheum..

[39] de Lange-Brokaar BJE (2016). Characterization of synovial mast cells in knee osteoarthritis: Association with clinical parameters. Osteoarthr. Cartil..

[40] Hua J, Sakamoto K, Nagaoka I (2002). Inhibitory actions of glucosamine, a therapeutic agent for osteoarthritis, on the functions of neutrophils. J. Leukoc. Biol..

[41] Tsuneyoshi Y (2012). Functional folate receptor beta-expressing macrophages in osteoarthritis synovium and their M1/M2 expression profiles. Scand. J. Rheumatol..

[42] Steinbeck MJ, Nesti LJ, Sharkey PF, Parvizi J (2007). Myeloperoxidase and chlorinated peptides in osteoarthritis: Potential biomarkers of the disease. J. Orthop. Res..

[43] Punzi L (2012). Coll2-1, Coll2-1NO2 and myeloperoxidase serum levels in erosive and non-erosive osteoarthritis of the hands. Osteoarthr. Cartil..

